# Tislelizumab uniquely binds to the CC′ loop of PD‐1 with slow‐dissociated rate and complete PD‐L1 blockage

**DOI:** 10.1002/2211-5463.13102

**Published:** 2021-02-16

**Authors:** Yuan Hong, Yingcai Feng, Hanzi Sun, Bo Zhang, Hongfu Wu, Qing Zhu, Yucheng Li, Tong Zhang, Yilu Zhang, Xinxin Cui, Zhuo Li, Xiaomin Song, Kang Li, Mike Liu, Ye Liu

**Affiliations:** ^1^ BeiGene Global Research BeiGene (Beijing) Co., Ltd. China

**Keywords:** anti‐PD‐1 antibody, BGB‐A317, epitope mapping, PD‐1, tislelizumab

## Abstract

Programmed cell death protein 1 (PD‐1), an immune checkpoint receptor expressed by activated T, B, and NK cells, is a well‐known target for cancer immunotherapy. Tislelizumab (BGB‐A317) is an anti‐PD‐1 antibody that has recently been approved for treatment of Hodgkin's lymphoma and urothelial carcinoma. Here, we show that tislelizumab displayed remarkable antitumor efficacy in a B16F10/GM‐CSF mouse model. Structural biology and Surface plasmon resonance (SPR) analyses revealed unique epitopes of tislelizumab, and demonstrated that the CC′ loop of PD‐1, a region considered to be essential for binding to PD‐1 ligand 1 (PD‐L1) but not reported as targeted by other therapeutic antibodies, significantly contributes to the binding of tislelizumab. The binding surface of tislelizumab on PD‐1 overlaps largely with that of the PD‐L1. SPR analysis revealed the extremely slow dissociation rate of tislelizumab from PD‐1. Both structural and functional analyses align with the observed ability of tislelizumab to completely block PD‐1/PD‐L1 interaction, broadening our understanding of the mechanism of action of anti‐PD‐1 antibodies.

## Graphical abstract

We evaluated the antitumor efficacy of tislelizumab (BGB‐A317), an approved anti‐PD‐1 antibody. Its critical epitopes were investigated by both structural biology and SPR studies, and the CC′ loop of PD‐1 was discovered to be a novel targetable region for anti‐PD‐1 antibodies. The unique epitopes and slow dissociation rate of tislelizumab contribute to its complete blocking activity to PD‐L1.

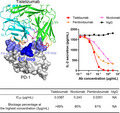

**Abbreviations**
i.p.: intraperitoneal
RU: responsive unit
SPR: surface plasmon resonance
TGI: tumor growth inhibition
V_H_: variable region of heavy chain
V_L_: variable region of light chain

Immune responses mediated by T cells are triggered when the antigenic peptide/HLA complexes on antigen‐presenting cell (APC) surface are recognized by T‐cell receptors (TCR). The regulation of these T‐cell‐mediated immune responses is balanced by antigen‐independent costimulatory or coinhibitory coreceptor signals [1, 2, 3]. T cells can recognize the tumor‐specific antigens presented on the surface of cancer cells, causing immune response against the cancer. However, cancer cells also develop some tumor‐induced immune suppression mechanisms to evade immunological recognition [4, 5]. One of the mechanisms of immune suppression is to upregulate the expression of immune suppressive molecules or their ligands or receptors, for example LAG‐3, galectin‐9/TIM‐3, CTLA4, and programmed death‐1 (PD‐1), inhibiting the activation of effector T cells, thus leading to poor response by the immune system to the cancer [6].

As a well‐known immune checkpoint molecule, PD‐1 is expressed in multiple types of immune cells, including activated T cells, B cells, certain dendritic cells (DCs), natural killer (NK) cells, and monocytes [7]. When binding to its natural ligands, PD‐1 ligand 1 (PD‐L1) or PD‐1 ligand 2 (PD‐L2), PD‐1 activates intracellular signaling pathways and inhibits the activation of immune cells [8, 9]. Previous studies have shown that immune suppression occurs in the tumor microenvironment while upregulating expression of PD‐1 and PD‐L1 in T cells and tumor cells, respectively [4, 5]. Thus, blocking the PD‐1 pathway has become a key direction to abolish immune suppression for immunotherapy, and the development of monoclonal antibodies targeting PD‐1 or PD‐L1 has been a research ‘hot spot’ of tumor immunotherapy in recent years [10, 11, 12, 13, 14, 15]. Two antibodies targeting PD‐1, pembrolizumab (Merck & Co., Inc., Kenilworth, NJ, USA) and nivolumab (Bristol‐Myers Squibb, New York, NY, USA), have been approved for treatment of bladder cancer, renal cell carcinoma, melanoma, Hodgkin's lymphoma, non‐small‐cell lung cancer, and other tumors by the US Food and Drug Administration (FDA) since 2014 [16]. Tislelizumab (BGB‐A317), an anti‐PD‐1 antibody, has been approved recently as a treatment for patients with classical Hodgkin's lymphoma who have failed at least one prior therapy or patients with locally advanced or metastatic urothelial carcinoma. Additionally, tislelizumab is being evaluated in global pivotal trials in a wide range of tumors, including esophageal squamous cell carcinoma, hepatocellular carcinoma, and non‐small‐cell lung cancer [17].

Previous structural biology studies on the complexes of PD‐1 with pembrolizumab or nivolumab have provided valuable information for understanding the binding mechanism of these two approved anti‐PD‐1 therapies [18, 19, 20]. In this study, we investigated the antitumor efficacy of tislelizumab and its binding kinetics to PD‐1, and then identified the key epitopes of tislelizumab by both structural biology study and surface plasmon resonance (SPR) analysis. Both the structural and functional studies revealed the unique interaction of tislelizumab with PD‐1 and provided insight into its mechanism of action by completely blocking the interaction of PD‐1 with PD‐L1. This work represents the first report to demonstrate the CC′ loop of PD‐1 as a targetable region for anti‐PD‐1 antibodies.

## Materials and methods

### Key materials

The hPD‐1 C57BL/6 transgenic mice used for *in vivo* efficacy evaluation were purchased from Biocytogen Co., Ltd. (Beijing, China). The antibodies pembrolizumab and nivolumab were purchased from Selleckchem (Houston, TX, USA), while tislelizumab was a gift from BI (Shanghai, China). HEK293F cells expressing recombinant PD‐1 or tislelizumab Fab proteins were obtained from Thermo Fisher (Shanghai, China).

### *In vivo* efficacy study

B16F10/GM‐CSF tumor cells (5 × 10^4^) were implanted subcutaneously in hPD‐1 C57BL/6 transgenic mice. On the day of implantation, mice were randomized into two groups and then treated with PBS (vehicle) or 10 mg·kg^−1^ of tislelizumab intraperitoneally (i.p.) once a week for 4 weeks. Tumor volume was measured twice weekly. All experiments involving animals were conducted based on the protocols approved by the Animal Care and Use Committee of BeiGene according to the guidelines of the Chinese Association for Laboratory Animal Sciences.

### Plasmid construction and site‐directed mutagenesis

The coding sequences of heavy and light chains of tislelizumab Fab were cloned into pMax vector, respectively, with a hexa‐His tag at the C terminus of heavy chain for purification. The DNA encoding the ectodomain of hPD‐1 (residues M1‐T170, including signal peptide, UniProt: Q15116) fusion with a TEV site and a human Fc tag was cloned into pMax vector. The plasmids of mutated PD‐1 were generated by a standard site‐directed mutagenesis method (NEB, Ipswich, MA, USA) with a pair of primers for each mutation.

### Protein expression and purification

Plasmids pMax‐heavy_chain and pMax‐light_chain of tislelizumab Fab were transiently cotransfected into HEK293F cells (Thermo Fisher) for protein expression. The supernatant was collected, and the tislelizumab Fab was purified by His tag affinity resin, followed by further purification with Superdex 200 (GE Healthcare, Shanghai, China) in a buffer containing 20 mm Tris and 150 mm NaCl (pH 8.0).

Plasmid pMax‐PD‐1 was transiently transfected into HEK293F cells (Thermo Fisher) for protein expression. The supernatant was collected, and the PD‐1 was purified by protein A, followed by treatment with TEV Protease to remove the Fc tag. Then, the Fc‐removed PD‐1 was further purified with Superdex 200 (GE Healthcare) in a buffer containing 20 mm Tris and 150 mm NaCl (pH 8.0). The mutated PD‐1 proteins were expressed and purified at similar condition as described above. The Fc tags of all PD‐1 proteins used for SPR analysis were not removed.

### Complex preparation and crystallization

The mammalian cell‐expressed WT PD‐1 protein and tislelizumab Fab fragment were mixed at a molar ratio of 1 : 1. The mixed sample was incubated for 30 min on ice and then purified by gel filtration in a buffer containing 20 mm Tris and 150 mm NaCl (pH 8.0). The complex peak was collected and concentrated to 30 mg·mL^−1^, followed by crystal screening using the vapor‐diffusion sitting‐drop method at 20 °C. Diffracting crystals were obtained in a condition consisting of 0.1 m citric acid, pH 4.0, 1 m LiCl, and 20% PEG6000.

### X‐ray data collection and structural determination

The crystals were harvested with Nylon loops and immersed in the reservoir solution supplemented with 20% glycerol for 10 s and then were flash‐cooled in liquid nitrogen. Diffraction data were collected at BL17U1, Shanghai Synchrotron Radiation Facility, and were processed with XDS program [21]. The phases were solved with program PHASER using structures of tislelizumab Fab fragment (our in‐house structure not shown in this study) and PD‐1 extracted from the complex structure of PD‐1 with nivolumab Fab (PDB: 5WT9) [20] as the molecular replacement searching models. Phenix.refine was used to perform rigid body, TLS, and restrained refinement against X‐ray data, followed by manual adjustment in COOT program and further refinement in Phenix.refine program [22, 23]. The atomic coordinates and structural factors of PD‐1/tislelizumab Fab complex have been deposited in the RCSB Protein Data Bank with accession number PDB: 7CGW.

### SPR analysis

Surface plasmon resonance analysis was performed at room temperature using a BIAcore 8K system with CM5 chips (GE Healthcare). For all measurements, an HBS‐EP plus buffer consisting of 10 mm HEPES, pH 7.4, 150 mm NaCl, 3 mm EDTA, and 0.005% (v/v) surfactant P20 was used as running buffer. The Fc‐tagged PD‐1 proteins were directly immobilized on the chip at a concentration of 150 nm. Gradient concentrations of antibody Fab (0, 1.56, 3.125, 6.25, 12.5, 25, 50, and 100 nm) were then flowed over the chip surface. After each cycle, the sensor surface was regenerated with 0.1 m glycine, pH 1.5. The binding kinetics were all analyzed with the software of BIA evaluation using a 1 : 1 steady‐state affinity model.

### P3Z assay

P3Z assay was carried out as previously described [24]. Briefly, HuT78/P3Z cells were preincubated with gradient concentrations of antibodies for 15 min prior to coculturing with HEK293/PD‐L1 cells in 96‐well plates (Costar, New York, NY, USA) containing complete RPMI 1640 media. The cells were incubated for 17 h at 37 °C. IL‐2 secretion in the supernatants of coculture was assayed by ELISA using a kit from eBioscience according to the manufacturer's instructions.

### Quantification and statistical analysis

Statistical analysis for the SPR and P3Z assays was analyzed with program Excel (Microsoft Corporation, Redmond, WA, USA) or program Prism (GraphPad Software, Inc, La Jolla, CA, USA).

## Results

### Tislelizumab shows remarkable antitumor efficacy in B16F10/GM‐CSF mouse model

**Fig. 1:**
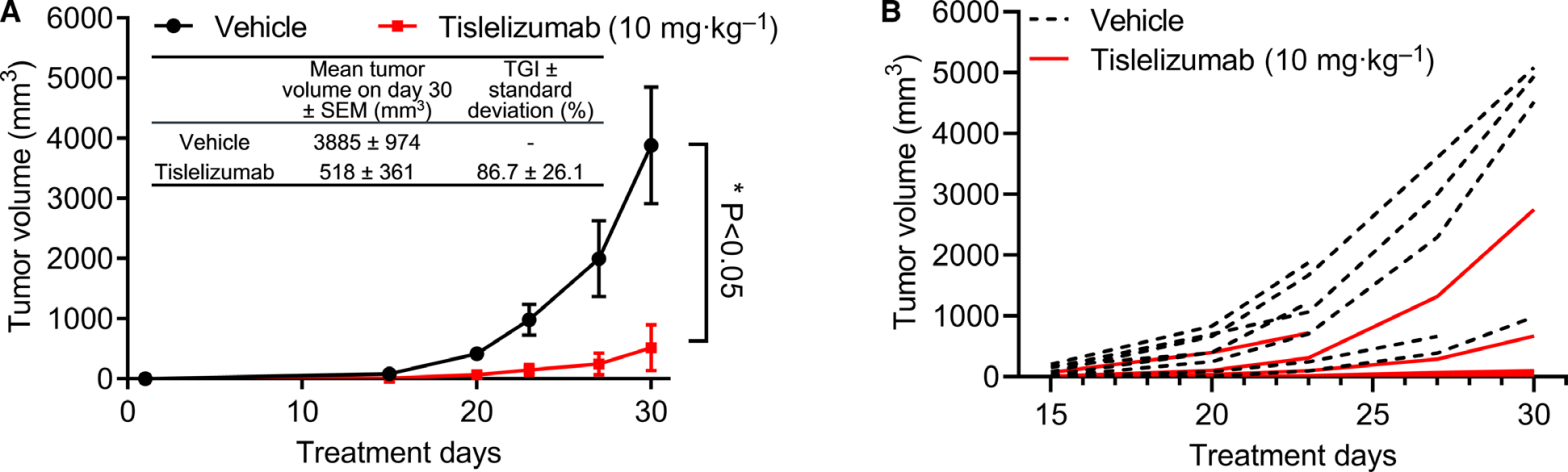
Antitumor efficacy of tislelizumab in B16F10/GM‐CSF hPD‐1 transgenic mouse model. (A) Mice bearing B16F10/GM‐CSF melanoma cells were treated i.p. with PBS (vehicle) or 10 mg·kg^−1^ of tislelizumab once a week. Tumor volume was measured twice weekly. Data are presented as mean tumor volume ± standard error of the mean (SEM), *n* = 8 mice in each group. *P < 0.05 by two‐tailed Student's *t*‐test. (B) Tumor volume of individual mice is presented for the PBS (vehicle) and tislelizumab treatment group. Shortened lines indicate mice that were found dead or euthanized according to study protocol (Table S2).

In this study, we evaluated the *in vivo* antitumor efficacy of tislelizumab in B16F10 melanoma‐bearing mouse model. B16F10 melanoma cells overexpressing GM‐CSF (B16F10/GM‐CSF; 5 × 10^4^) were implanted subcutaneously in hPD‐1 C57BL/6 transgenic mice. PBS (vehicle) or tislelizumab (dose of 10 mg·kg^−1^) was injected i.p. once a week, and tumor volumes were monitored twice a week. It was noted that five out of eight mice responded very well to tislelizumab treatment. The overall tumor growth of the tislelizumab‐treated mice was significantly reduced in comparison with the vehicle group, resulting in approximate 86.7% tumor growth inhibition (TGI) with *P* < 0.05 (Fig. 1). This result revealed that tislelizumab exerted remarkable antitumor efficacy.

### The dissociation rate of tislelizumab from PD‐1 is much slower than that of pembrolizumab and nivolumab

**Fig. 2:**
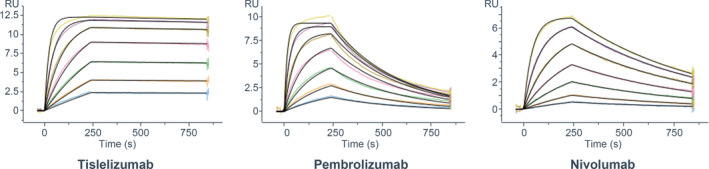
SPR assay characterization of the binding affinity of tislelizumab, pembrolizumab, and nivolumab to PD‐1. The data presented here are representatives of three independent experiments with similar results. RU, responsive unit.

In order to compare the binding affinity of tislelizumab, pembrolizumab, and nivolumab to PD‐1, the binding kinetics of the three antibodies were investigated by SPR. As shown in Fig. 2 and Table 1, the association rate (*K*_a_) of tislelizumab (4.24 × 10^5^ m ^−1·^s^−1^) with PD‐1 equals approximately twofold of that of nivolumab (2.27 × 10^5^ m ^−1·^s^−1^) and one half of that of pembrolizumab (9.54 × 10^5^ m ^−1·^s^−1^). However, the dissociation rate (*K*_d_) of tislelizumab (4.82 × 10^−5^ s^−1^) is about 30‐fold slower than that of nivolumab (1.57 × 10^−3^ s^−1^) and 80‐fold less than of pembrolizumab (3.88 × 10^−3^ s^−1^), respectively, resulting in a 35‐ to 60‐fold higher target affinity of tislelizumab compared with the other two antibodies. At the same time, the *t*_1/2_ of tislelizumab is 30‐ to 80‐fold longer than that of pembrolizumab and nivolumab (Table 1). These results indicate that the high affinity of tislelizumab to PD‐1 mainly results from its extremely slow dissociation rate from PD‐1.

**Table 1:** Binding kinetics of tislelizumab to WT PD‐1 and its comparison with pembrolizumab and nivolumab. Data are presented as mean ± SEM of results of three independent experiments.

Sample	*k*_a_ (m ^−1·^s^−1^)	*k*_d_ (s^−1^)	*K*_D_ (m)	*t*_1/2_ (min)
Tislelizumab Fab	4.24 ± 0.094 × 10^5^	4.82 ± 0.66 × 10^−5^	1.14 ± 0.15 × 10^−10^	248 ± 32
Pembrolizumab Fab	9.54 ± 1.2 × 10^5^	3.88 ± 0.29 × 10^−3^	4.07 ± 0.25 × 10^−9^	3.0 ± 0.23
Nivolumab Fab	2.27 ± 0.063 × 10^5^	1.57 ± 0.005 × 10^−3^	6.92 ± 0.17 × 10^−9^	7.3 ± 0.02

### Unique epitopes of tislelizumab are identified by structural and SPR analyses

**Fig. 3:**
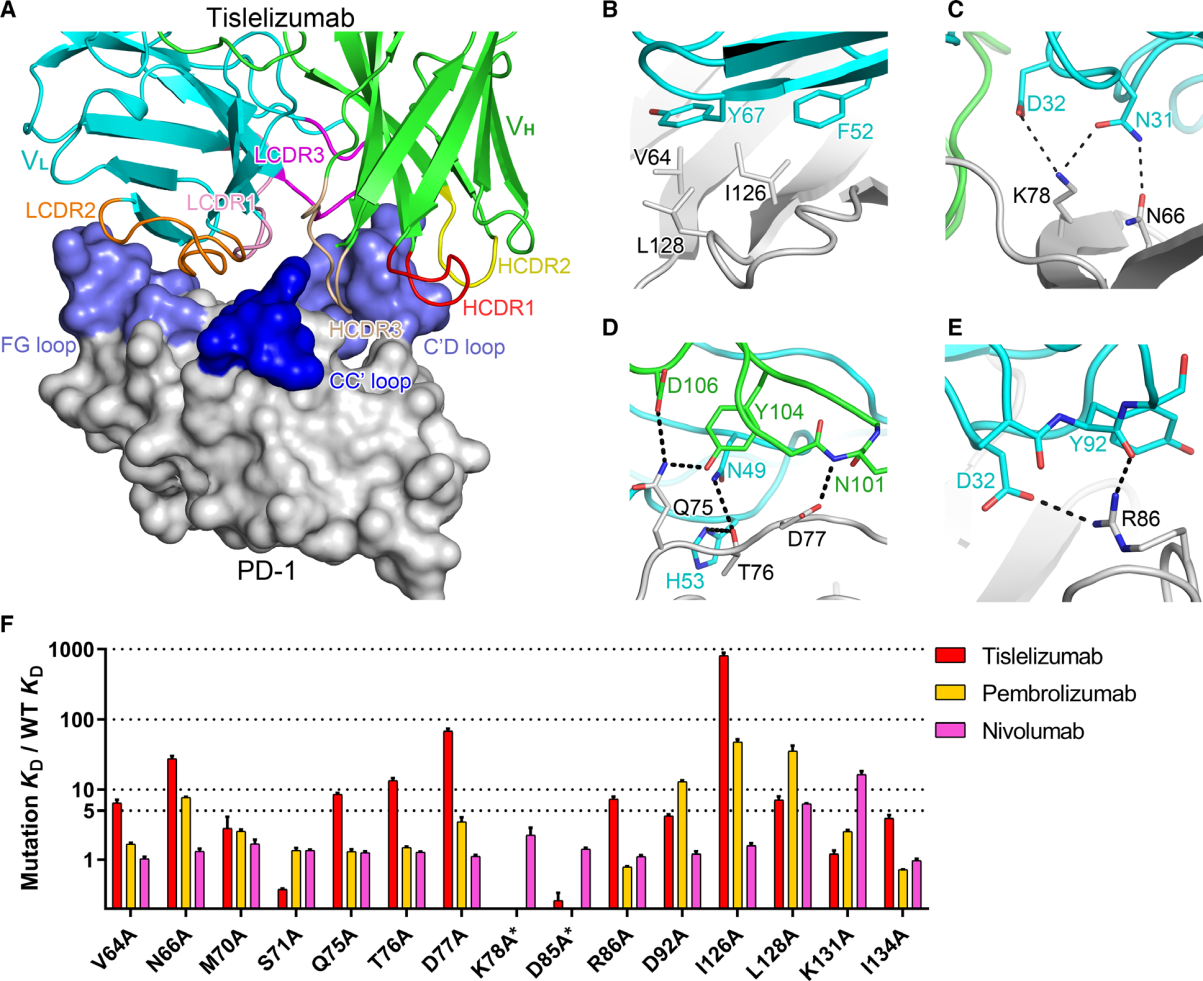
Detailed interactions between tislelizumab and PD‐1 and summary of SPR study. (A) The tislelizumab Fab is shown as ribbon (heavy chain, green; light chain, cyan), and PD‐1 is shown as surface representation (gray). CC′, C′D, and FG loops of PD‐1 are highlighted in blue or light blue. The HCDR1, HCDR2, HCDR3, LCDR1, LCDR2, and LCDR3 of tislelizumab are colored in red, yellow, light golden, pink, orange, and magenta, respectively. (B–E) The PD‐1, V_H_, and V_L_ of tislelizumab are colored in gray, green, and cyan, respectively. Hydrogen bonds and a salt bridges are indicated as black dashed lines. (F) The SPR profile of tislelizumab, pembrolizumab, and nivolumab against PD‐1 mutants. Data are presented as mean with SEM, *n* = 3. *, the *K*_D_ shift folds of tislelizumab and pembrolizumab against mutant K78A, as well as those of pembrolizumab against D85A, could not be accurately calculated due to no significant binding signal being detected during SPR analysis.

The remarkable antitumor activity of tislelizumab and its extremely slow dissociation property prompted our curiosity on the binding mechanism and epitopes of tislelizumab. To address this question, we cocrystallized the complex of tislelizumab Fab fragment with the ectodomain of PD‐1, both of which were expressed in a mammalian cell system and solved the complex structure at a resolution of 3.2 Å (Table S1). The complex structure reveals that tislelizumab utilizes four of six complementarity‐determining regions (CDRs) to interact with PD‐1 (Fig. 3A) with a total buried surface of 2014 Å^2^. All three CDR loops in the variable region of light chain (V_L_) and CDR3 loop in the variable region of heavy chain (V_H_) are involved in interaction with PD‐1.

**Fig. 4:**
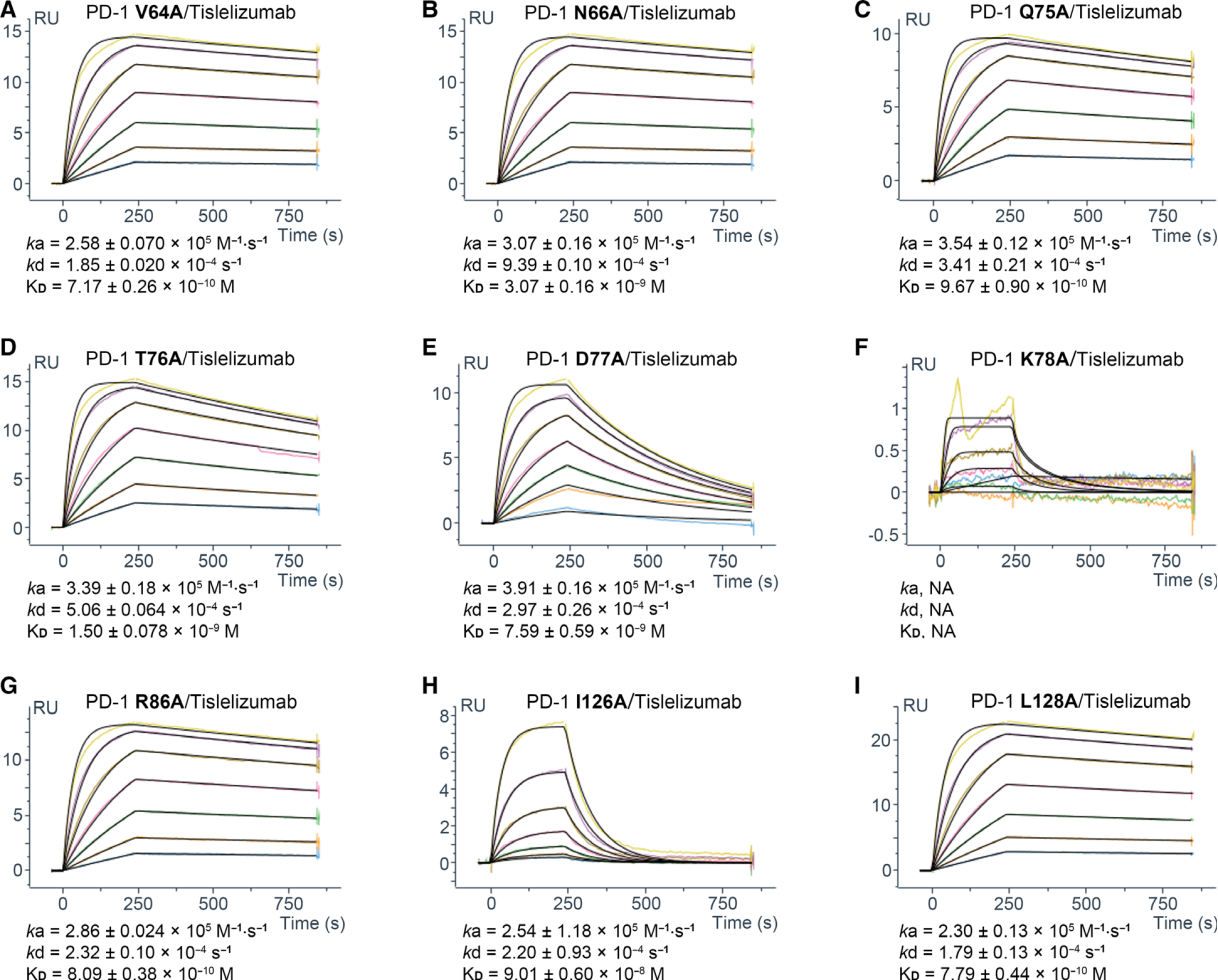
The SPR profile of tislelizumab against specific PD‐1 mutations in the epitope study. The data presented here are representatives of three independent experiments with similar results. The binding kinetics parameters are presented as the mean ± SEM, *n* = 3. NA, the binding kinetics of tislelizumab against mutant K78A could not be accurately calculated due to no significant binding signal being detected during SPR analysis. RU, responsive unit.

Based on this structural information, we carried out mutagenesis on a series of PD‐1 residues observed to potentially contribute to the PD‐1/tislelizumab interaction, including Val64, Asn66, Met70, Ser71, Gln75, Thr76, Asp77, Lys78, Asp85, Arg86, Asp92, Ile126, Leu128, Lys131, and Ile134. The binding affinities of these mutated PD‐1 proteins to tislelizumab were evaluated by SPR compared with WT PD‐1. Meanwhile, pembrolizumab and nivolumab were also analyzed via SPR with the same PD‐1 mutant proteins. The binding affinity of several mutated PD‐1 proteins to tislelizumab significantly decreased compared with WT (Table 1 and Fig. 4A–I). Specifically, the *K*_D_ shift folds (*K*_D_ of mutation/*K*_D_ of WT) of V64A, Q75A, R86A, and L128A are more than 5, while the shift folds of N66A, T76A, and D77A range from 13 to 68, and that of I126A is nearly 800 (Fig. 3F and Table 2). The SPR results of other mutants with pembrolizumab and nivolumab are also summarized in Figs 3F and 4 and Table 2.

**Table 2:** Summary of *K*_D_ shift folds of PD‐1 mutants compared with WT. Data are presented as mean value ± SEM of results of three independent experiments. NA, the *K*_D_ shift folds of tislelizumab and pembrolizumab against mutant K78A, as well as those of pembrolizumab against D85A, could not be accurately calculated due to no significant binding signal being detected during SPR analysis.

PD‐1 mutants	*K*_D_ shift folds of tislelizumab	*K*_D_ shift folds of pembrolizumab	*K_D_* shift folds of nivolumab
V64A	6.5 ± 0.67	1.7 ± 0.081	1.0 ± 0.075
N66A	28 ± 2.3	7.7 ± 0.18	1.3 ± 0.12
M70A	2.8 ± 1.3	2.5 ± 0.16	1.7 ± 0.26
S71A	0.4 ± 0.01	1.4 ± 0.11	1.4 ± 0.046
Q75A	8.6 ± 0.34	1.3 ± 0.094	1.3 ± 0.057
T76A	13 ± 1.1	1.5 ± 0.032	1.3 ± 0.029
D77A	68 ± 5.5	3.5 ± 0.53	1.1 ± 0.044
K78A	NA	NA	2.2 ± 0.60
D85A	0.3 ± 0.074	NA	1.4 ± 0.073
R86A	7.3 ± 0.67	0.8 ± 0.013	1.1 ± 0.038
D92A	4.2 ± 0.24	13 ± 0.63	1.2 ± 0.11
I126A	809 ± 78	48 ± 3.9	1.6 ± 0.12
L128A	7.1 ± 0.90	36 ± 6.5	6.3 ± 0.11
K131A	1.2 ± 0.15	2.5 ± 0.14	16 ± 2.0
I134A	3.9 ± 0.43	0.7 ± 0.005	1.0 ± 0.063

The detailed interactions between tislelizumab and residues V64, N66, Q75, T76, D77, R86, I126, and L128 of PD‐1 are shown in Fig. 3B–E. The side chain of I126_PD‐1_ forms a hydrophobic interaction with F52 in the tislelizumab light chain (F52_LC_), and V64_PD‐1_ and L128_PD‐1_ interact with Y67_LC_ (Fig. 3B). In addition, a number of hydrogen bonds between N66_PD‐1_ and N31_LC_, Q75_PD‐1_ and Y104_HC_, Q75_PD‐1_ and D106_HC_, T76_PD‐1_ and N49_LC_, T76_PD‐1_ and H53_LC_, D77_PD‐1_ and N101_HC_, and R86_PD‐1_ and Y92_LC_, and a salt bridge between R86_PD‐1_ and D32_LC_ can further stabilize the PD‐1/tislelizumab complex (Fig. 3C–E). Besides, K78_PD‐1_ forms a hydrogen bond with N31_LC_ and a salt bridge with D32_LC_ (Fig. 3C). The K78A mutation in PD‐1 abolishes the binding of PD‐1 to tislelizumab (Fig. 4F). In conclusion, residues V64, N66, Q75, T76A, D77, K78, R86, I126, and L128 of PD‐1 were identified as key epitopes of tislelizumab by structural biology and experimentally confirmed by SPR analysis. Notably, mutations Q75A, T76A, D77A, and R86A only significantly affect the binding of tislelizumab rather than pembrolizumab and nivolumab (Fig. 3F and Table 2). Thus, residues Q75, T76, D77, and R86 of PD‐1 were identified as unique binding epitopes for tislelizumab compared with pembrolizumab and nivolumab.

### Tislelizumab is the first anti‐PD‐1 antibody targeting the CC′ loop of PD‐1

**Fig. 5:**
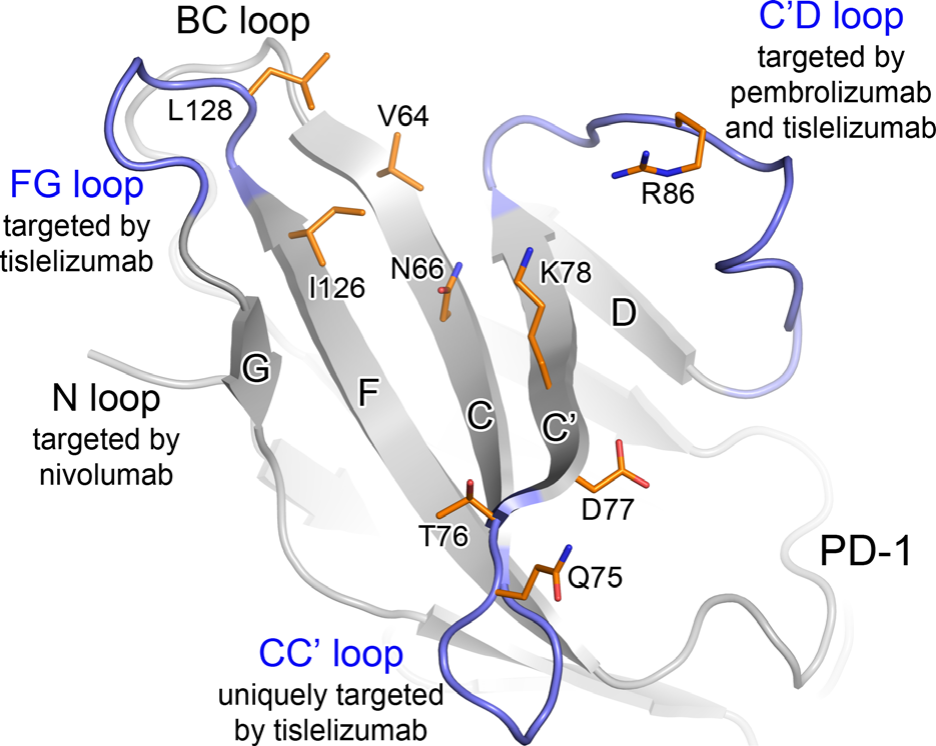
The key epitopes of tislelizumab. The capital characters C, C′, D, F, and G represent the β‐strands C, C′, D, F, and G of PD‐1, respectively. The CC′, C′D, and FG loops of PD‐1 or key epitopes of tislelizumab are highlighted in blue or orange colors, respectively.

Notably, three of four unique epitopes, Q75, T76, and D77, are located at the CC′ loop and adjacent area of PD‐1 (Fig. 5). The C′D or FG loops have been reported to dominate binding by several antibodies [18, 19, 25, 26, 27, 28]. However, the CC′ loop, to our knowledge, was never reported to bind with any anti‐PD‐1 antibody in previous publications.

The conformational change within the CC′ loop is considered to be essential for PD‐L1 binding to this loop [29, 30]. Thus, it is reasonable that an antibody targeting this CC′ loop could effectively abrogate PD‐L1 binding to PD‐1. However, no therapeutic anti‐PD‐1 antibodies were previously reported to bind to this loop. In the PD‐1/tislelizumab complex, the CC′ loop inserts into the space between HCDR3 and LCDR2, forming several hydrogen bonds between tislelizumab and PD‐1 (Fig. 3A,D). Thus, this is the first time a therapeutic antibody is reported to interact with the CC′ loop of PD‐1, supplying a direct evidence that the CC′ loop is an effective target region for therapeutic anti‐PD‐1 antibody.

### Tislelizumab completely blocks PD‐L1 by competitive binding to PD‐1

**Fig. 6:**
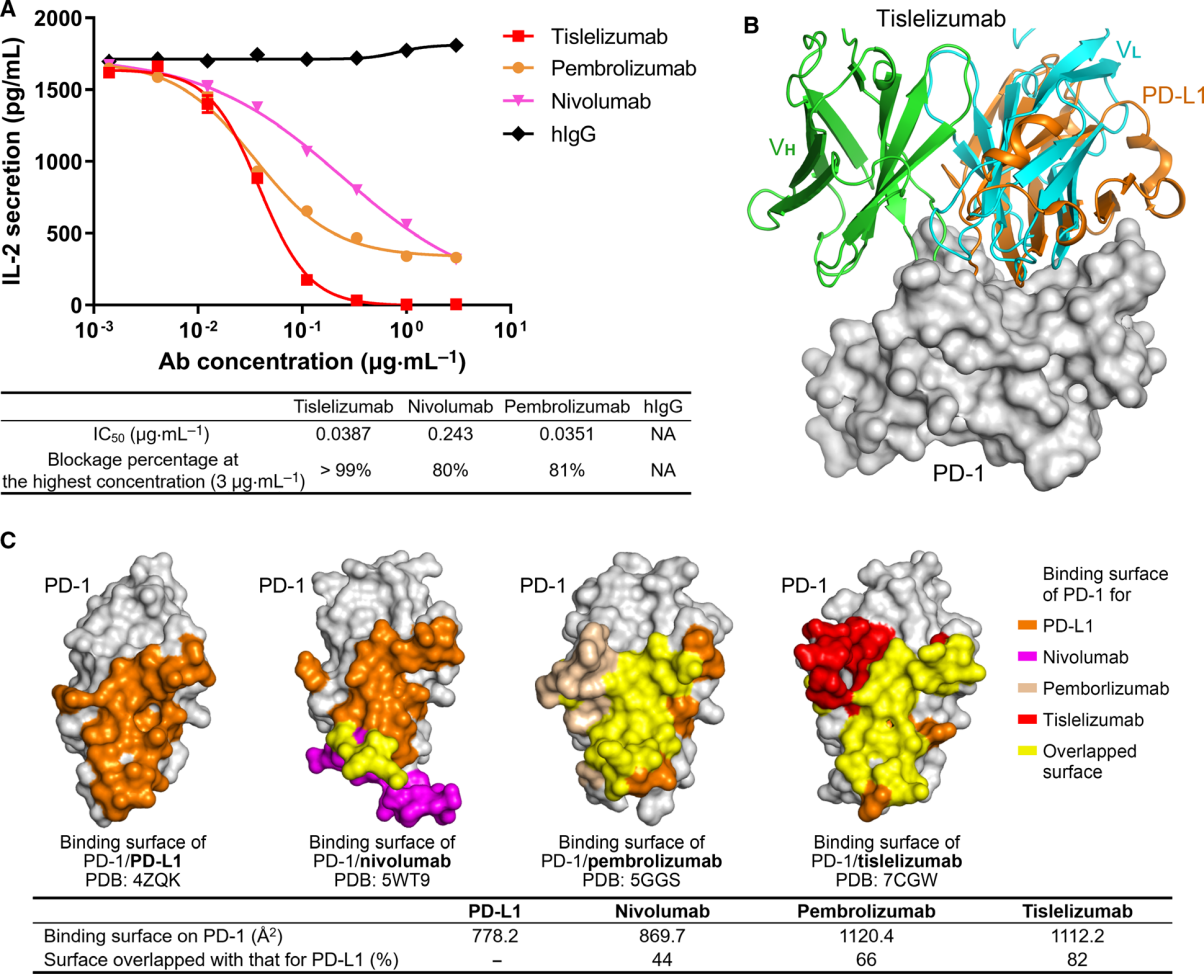
Complete PD‐L1 blockage of tislelizumab. (A) The PD‐L1 blockage activity of tislelizumab in P3Z assay in comparison with pembrolizumab and nivolumab. HuIgG was used as negative control. (B) Tislelizumab occupies the position of PD‐L1 harboring PD‐1. The heavy chain and light chain of V_H,_ tislelizumab Fab, PD‐L1, and PD‐1 are colored in green, cyan, orange, and gray, respectively. (C) Binding surface comparison between PD‐L1, nivolumab, pembrolizumab, and tislelizumab. Surface of PD‐1 molecule colored in orange, magenta, light golden, and red is the binding surface for PD‐L1, nivolumab, pembrolizumab, and tislelizumab, respectively, while the surface colored in yellow marks the overlap area with the PD‐L1/PD‐1 interface.

To evaluate the efficiency of anti‐PD‐1 antibodies to block PD‐L1 binding to PD‐1, we employed a cellular P3Z assay [24] to quantify the PD‐L1 blocking activity of the anti‐PD‐1 antibodies. This signaling assay uses a two‐cell coculture system, including the signal sensor cell, HuT78/P3Z, that expresses the chimeric PD‐1 receptor and the signal sending cell, HEK293/PD‐L1. The engagement of chimeric PD‐1 receptor with PD‐L1 leads to activation, instead of inhibition, of the sensor cell to secrete IL‐2. As shown in Fig. 6, both pembrolizumab and nivolumab showed partial PD‐L1 blocking activity, while tislelizumab completely blocked PD‐L1 binding to PD‐1 in the measured concentration range (Fig. 6A).

PD‐L1 binds to the front β‐sheet face of IgV domain of PD‐1 [29], and the binding of tislelizumab to PD‐1 also occurs to the same face (Fig. 3A). Superimposition of the tislelizumab/PD‐1 complex structure with the PD‐1/PD‐L1 complex structure (PDB: 4ZQK) reveals competitive binding of tislelizumab with PD‐L1. The competitive binding of tislelizumab mainly depends on the V_L_, which directly occupies the position of PD‐L1 harboring to PD‐1 (Fig. 6B). The detailed analysis of the binding surface on PD‐1 reveals that tislelizumab overlaps with approximately 80% of the binding area of PD‐L1 to PD‐1, significantly larger than the overlapping binding surfaces of nivolumab and pembrolizumab with the PD‐L1/PD‐1 interface (Fig. 6C). These findings suggest that tislelizumab likely provides maximum steric abrogation to PD‐L1 in its binding to PD‐1, aligning with the complete PD‐L1 blockage by tislelizumab in P3Z assay.

## Discussion

In this study, we evaluated the *in vivo* efficacy of tislelizumab in B16F10/GM‐CSF mouse model, demonstrating its remarkable antitumor activity. SPR analysis showed that tislelizumab has a much slower dissociation rate from PD‐1 and a longer *t*_1/2_ than both pembrolizumab and nivolumab. These findings prompted us to investigate the binding mechanism and epitopes of tislelizumab. To address this question, we solved the cocrystal structure of PD‐1 with tislelizumab Fab. Guided by the structure, a series of PD‐1‐mutated proteins were generated and subjected to SPR analysis. Both the structural and functional studies revealed the key epitopes of tislelizumab and provided insight into its mechanism of action by blocking the interaction of PD‐1 with PD‐L1.

**Fig. 7:**
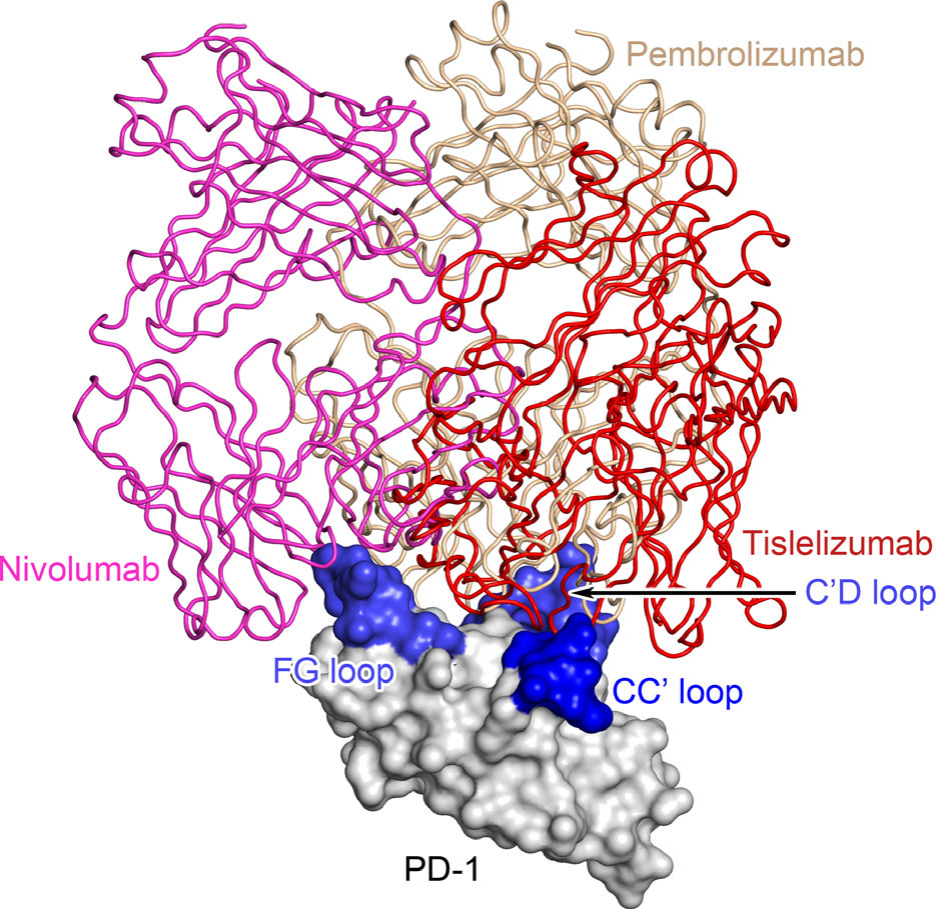
Distinct binding orientation of tislelizumab compared with pembrolizumab and nivolumab. Superimposition of PD‐1/tislelizumab complex with that of pembrolizumab (PDB: 5GGS) and nivolumab (PDB: 5WT9). PD‐1, tislelizumab, pembrolizumab, and nivolumab are colored in gray, red, light golden, and magenta, respectively. The CC′, C′D, and FG loops of PD‐1 are highlighted in blue.

The superposition of complex structures of PD‐1 with tislelizumab and pembrolizumab reveals that they bind to PD‐1 in different orientations (Fig. 7). In the PD‐1/pembrolizumab complex, the C′D loop of PD‐1 intrudes into the groove formed by the CDR loops of pembrolizumab [18, 19, 25], while the CC′, C′D, and FG loops of PD‐1 surround the HCDR3 and all three LCDR loops in the PD‐1/tislelizumab complex (Fig. 3A). The detailed structural analysis and SPR results indicated that Q75A, T76A, and D77A mutations at the CC′ loop and adjacent area of PD‐1 significantly reduce the binding affinity to tislelizumab, but show much less effect on binding to pembrolizumab (Fig. 3F and Table 2), implying that the CC′ loop of PD‐1 plays a pivotal role in binding with tislelizumab, different from pembrolizumab. D85A, a mutation in the C′D loop of PD‐1, completely disrupted PD‐1 binding to pembrolizumab in the SPR experiment (Fig. S1I), which is consistent with a previous report [31]. However, D85A does not affect the binding affinity between PD‐1 and tislelizumab (Fig. S1I and Table 2). By contrast, the R86A mutation at the same C′D loop showed no impact on PD‐1 binding to pembrolizumab, but caused sevenfold affinity decrease on that of tislelizumab (Table 2). SPR results of these two mutations indicate that, while both antibodies interact with the C′D loop of PD‐1, they target different epitopes.

On the other hand, structural analysis showed that the binding surface of tislelizumab on PD‐1 is quite different from that of nivolumab, with only a few overlapping residues. A comparative study between complex structures of PD‐1 with tislelizumab and nivolumab also reveals that the two antibodies bind to PD‐1 in two different orientations (Fig. 7). However, in our SPR results, those PD‐1 mutations (V64A, N66A, Q75A, T76A, D77A, R86A, and I126A) that significantly reduce the binding affinity to tislelizumab are not critical epitopes for nivolumab (Fig. 3F and Table 2).

Taken together, the structural analysis and SPR results demonstrate that tislelizumab has distinctive binding orientation to PD‐1 and unique epitopes compared with pembrolizumab and nivolumab. From the SPR analysis summarized in Table 2, it is concluded that Gln75, Thr76, Asp77, and Arg86 of PD‐1 are unique binding epitopes for tislelizumab compared with pembrolizumab and nivolumab. Notably, residues Gln75, Thr76, and Asp77 are all located at the CC′ loop and its adjacent area of PD‐1.

It has been previously reported that pembrolizumab binds to the C′D loop of PD‐1, while nivolumab mainly binds to the N loop [18, 19, 20, 25]. Recently, Liu *et al*. [27] reported that toripalimab, an antibody generated by Shanghai Junshi Bioscience, binds to the FG loop of PD‐1. Besides, several preclinical stage anti‐PD‐1 antibodies were reported to bind with the BC, C′D, or FG loops [26, 28, 32]. All the four loops referred above are far away from the CC′ loop in PD‐1 (Fig. 5). In contrast to all of the reported anti‐PD‐1 antibodies, tislelizumab is the first antibody that interacts with the CC′ loop of PD‐1.

The CC′ loop of PD‐1 is considered to play a critical role in the binding of PD‐1 to PD‐L1, and it undergoes a significant conformational change and transforms from an open conformation to a closed one after binding with PD‐L1 [29]. It has been reported that this conformational change in the CC′ loop could stabilize the interaction of PD‐1 with PD‐L1 and influence their binding affinity [29, 30]. As the mutations at the CC′ loop of PD‐1 significantly accelerated the off‐rate of tislelizumab and reduced its binding affinity in SPR (Fig. 4 and Table 1), we also speculate that the direct binding to the CC′ loop might contribute to the high affinity and slow off‐rate of tislelizumab. Taken together, the unique epitopes on the CC′ loop of PD‐1 contribute to the observation that tislelizumab completely blocks PD‐1/PD‐L1 interaction, while both nivolumab and pembrolizumab only show partial PD‐L1 blocking activity in the cellular assay (Fig. 6A).

## Conclusion

In summary, we report the remarkable antitumor efficacy of tislelizumab in tumor model and key epitopes of tislelizumab identified by both structural biology and SPR analysis. Distinct from all currently approved anti‐PD‐1 antibodies, tislelizumab uniquely binds to the CC′ loop of PD‐1, which is the first time to be reported to interact with an anti‐PD‐1 antibody. In addition, tislelizumab displays complete blocking activity to PD‐L1 with extremely slow dissociation rate from PD‐1, and its binding surface largely overlaps with that of PD‐L1. All these findings facilitate our understanding of the binding mechanisms of tislelizumab and provide a novel targetable region for the development of anti‐PD‐1 antibodies for tumor immunotherapy.

## Conflict of interest

The authors declare no conflict of interest.

## Author contributions

TZ, KL, ML, and YL conceived and designed this project. YH and YF performed X‐ray crystallography. HS analyzed the structure. BZ carried out SPR assays. HW expressed and purified PD‐1 mutant proteins. TZ, YL, and QZ designed and performed the P3Z assay. YZ, XC, ZL, and XS conducted the *in vivo* efficacy study. YH, HS, and YL wrote the manuscript. YL supervised this study.

## Supporting information


**Fig. S1.** The SPR profile of tislelizumab, pembrolizumab and nivolumab against PD‐1 mutants. The data presented here are representatives of three independent experiments with similar results. RU, responsive units.
**Table S1.** Data collection and refinement statistics.
**Table S2.** The original tumor measurement data of the *in vivo* efficacy study.

## Data Availability

The atomic coordinates and structural factors of PD‐1/tislelizumab Fab complex have been deposited at the RCSB Protein Data Bank with accession number PDB: 7CGW.
