# Supplementary material for: Tislelizumab uniquely binds to the CC′ loop of PD‐1 with slow‐dissociated rate and complete PD‐L1 blockage
Source: FEBS Open Bio. 2021 Feb 16;11(3):782–92. doi: 10.1002/2211-5463.13102 (PMC7931243; doi:10.1002/2211-5463.13102)
Supplement: Supplementary file 1 — Fig. S1. The SPR profile of tislelizumab, pembrolizumab and nivolumab against PD‐1 mutants. The data presented here are representatives of three independent experiments with similar results. RU, responsive units. Table S1. Data collection and refinement statistics. Table S2. The original tumor measurement data of the in vivo efficacy study. [file FEB4-11-782-s001.pdf]

Tislelizumab uniquely binds to the CC' loop of PD-1 with slow-dissociated rate and complete PD-L1 blockage

Yuan Hong†, Yingcai Feng†, Hanzi Sun, Bo Zhang, Hongfu Wu, Qing Zhu, Yucheng Li, Tong Zhang, Yilu Zhang, Xinxin Cui, Zhuo Li, Xiaomin Song, Kang Li, Mike Liu and Ye Liu*

BeiGene Global Research, BeiGene (Beijing) Co., Ltd., Beijing 102206, P.R.China;

† These authors contributed equally to this work

* Correspondence
Ye Liu, BeiGene Global Research, BeiGene (Beijing) Co., Ltd., Beijing 102206, P.R.China
Tel.: +86-010-58254385
E-mail: ye.liu@beigene.com

Supporting information

Table S1. Data collection and refinement statistics

	7CGW
**Data collection**	
Beamline	BL17U1, SSRF
Space group	C 2 2 21
Cell dimensions (Å)	a=142.64 b=145.57 c=175.55
Angles (°)	α=90.00 β=90.00 γ=90.00
Resolution (Å)	101.88-3.20 (3.37-3.20) *
Total number of reflections	346745 (49703)
Number of unique reflections	30470 (4399)
Completeness (%)	99.9 (99.8)
Average redundancy	11.4 (11.3)
Rmerge	0.155 (1.093)
I/sigma (I)	11.8 (2.4)
Wilson B factor (Å)	95.76
**Refinement**	
Resolution (Å)	101.88-3.20
Number of reflections	30423
rmsd bond lengths (Å)	0.006
rmsd bond angles (°)	1.095
R_{work} (%)	21.79
R_{free} (%)	25.40
Average B-factors of protein	103.9
Ramachandran plot (%)	
Favored	94.53
Allowed	5.19
Outliers	0.28

* Values in parentheses refer to the highest resolution shell.

Table S2. The original tumor measurement data of the *in vivo* efficacy study

Treatment	Mouse ID	Individual animal's tumor volume (mm^3)					
		**Day 1**	**Day 15**	**Day 20**	**Day 23**	**Day 27**	**Day 30**
**Vehicle**	# 1	-	81	666	1672	3609	5088
	# 2	-	34	400	1224	-	-
	# 3	-	7	253	708	2296	4520
	# 4	-	0	89	247	668	-
	# 5	-	0	12	98	394	986
	# 6	-	176	399	-	-	-
	# 7	-	151	702	1067	3010	4943
	# 8	-	208	839	1878	-	-
**Tislelizumab (10 mg/kg)**	# 9	-	7	5	14	39	76
	# 10	-	0	0	0	0	0
	# 11	-	0	3	18	75	103
	# 12	-	0	35	100	294	673
	# 13	-	74	405	724	-	-
	# 14	-	8	108	310	1324	2753
	# 15	-	0	0	0	0	0
	# 16	-	0	0	0	8	25

Data unavailable after the Day 20 indicated mice that were found dead or euthanized according to study protocol.

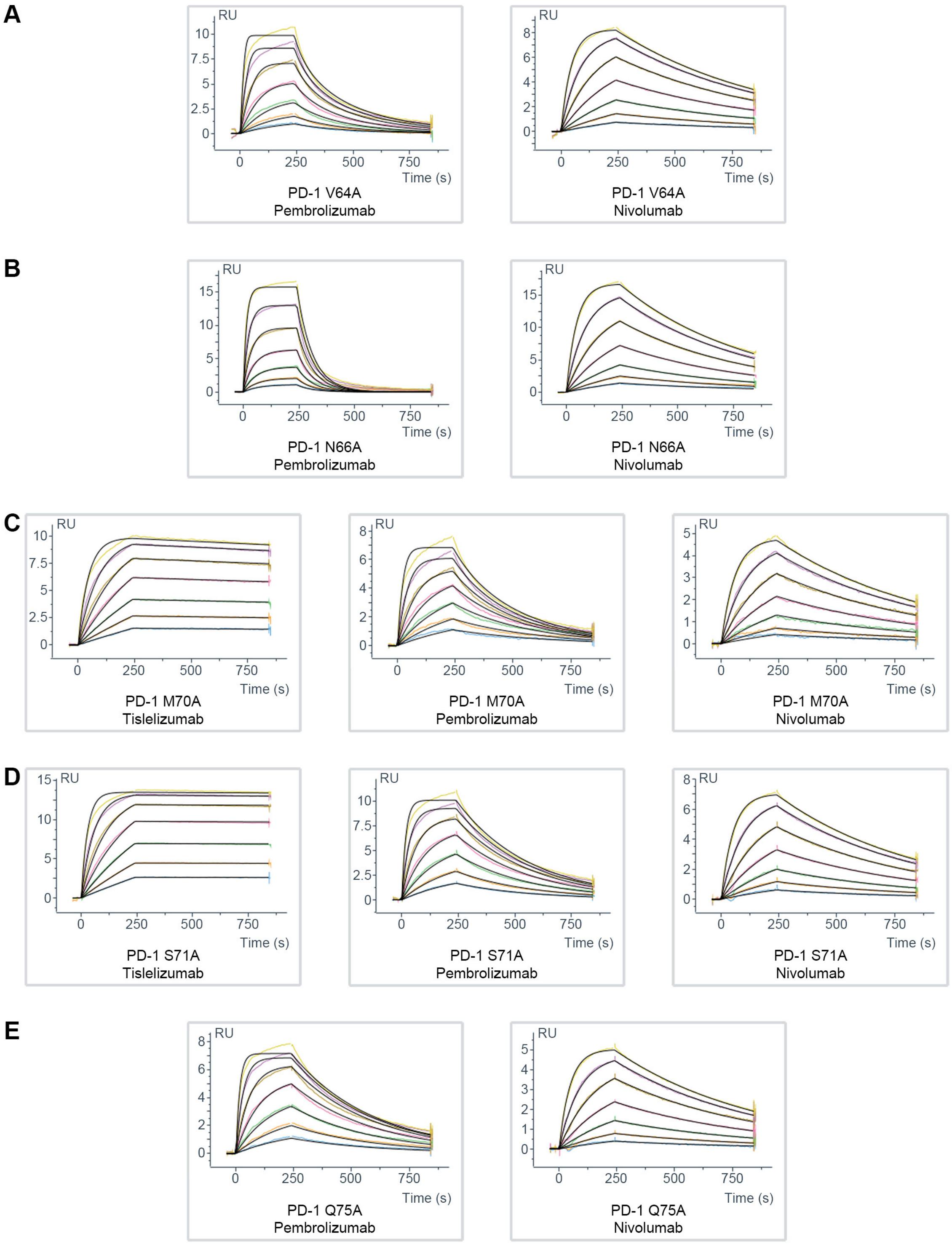

Figure S1. Continued.

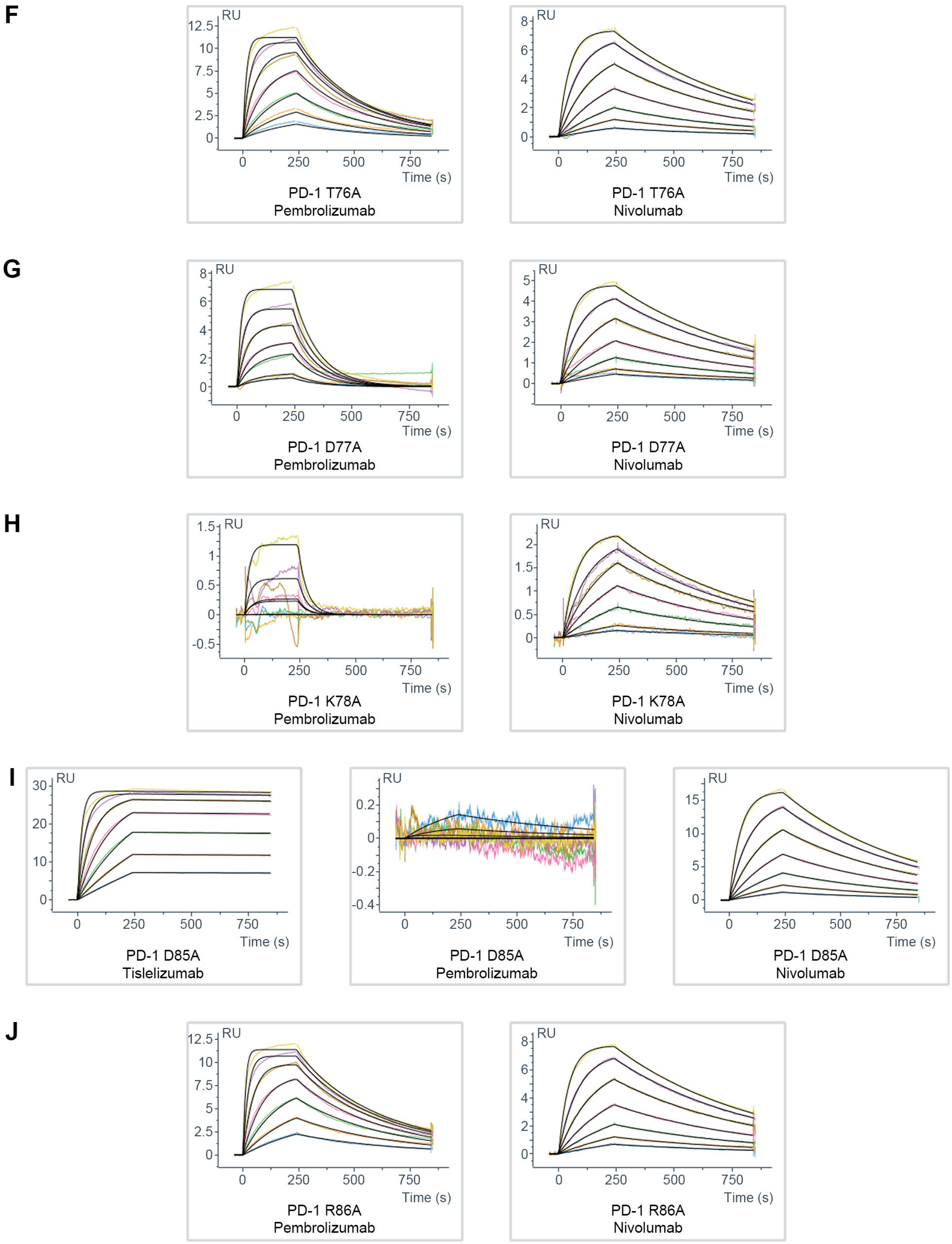

Figure S1. Continued.

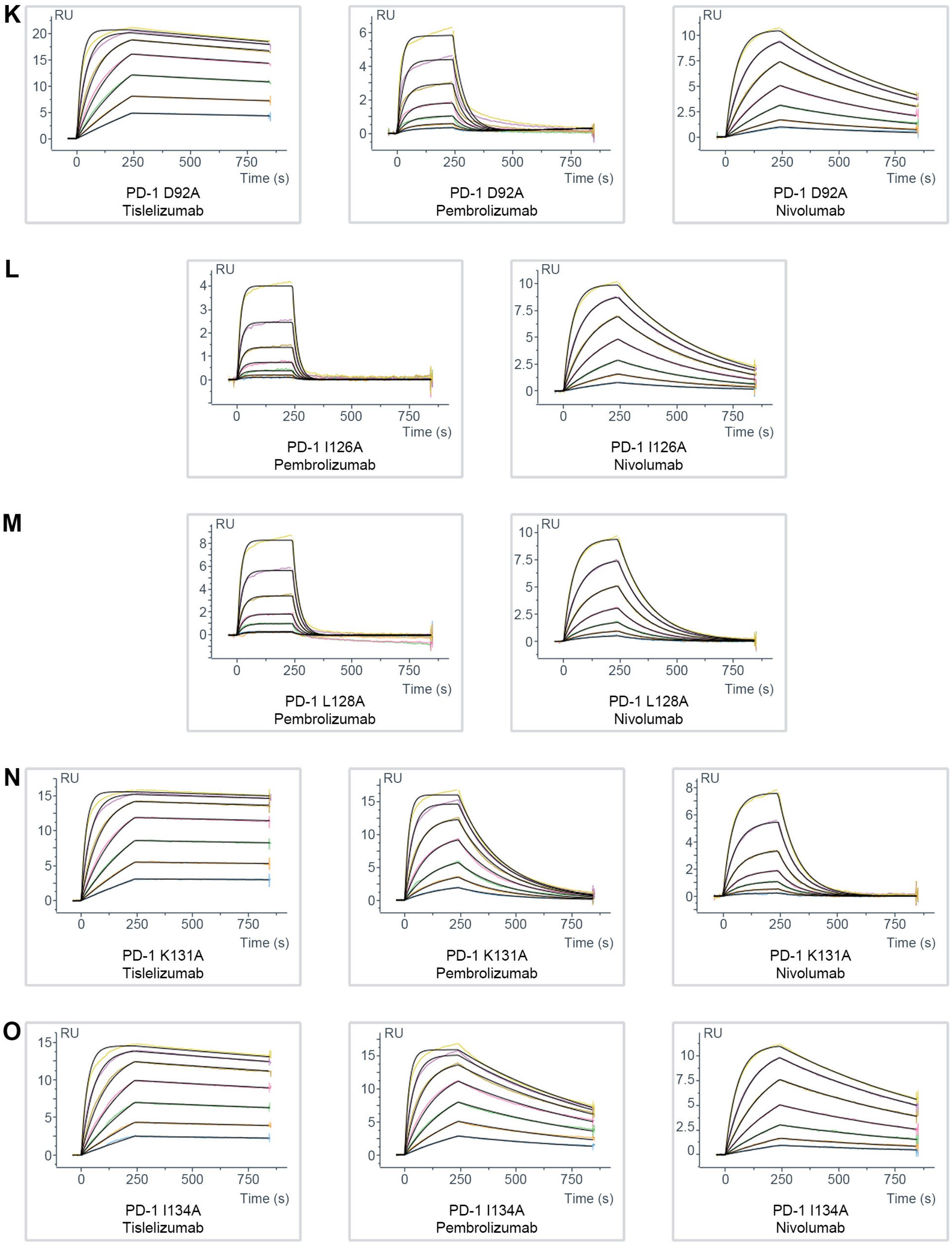

Figure S1. The SPR profile of tislelizumab, pembrolizumab and nivolumab against PD-1 mutants. The data presented here are representatives of three independent experiments with similar results. Abbreviations: RU, responsive units.
